# Pyrrolizidine Alkaloids: The Botanical Origin of Pollen Collected during the Flowering Period of *Echium vulgare* and the Stability of Pyrrolizidine Alkaloids in Bee Bread

**DOI:** 10.3390/molecules24122214

**Published:** 2019-06-13

**Authors:** Christina Kast, Verena Kilchenmann, Hans Reinhard, Katharina Bieri, Otmar Zoller

**Affiliations:** 1 Agroscope, Swiss Bee Research Centre, Schwarzenburgstrasse 161, 3003 Bern, Switzerland; verena.kilchenmann@agroscope.admin.ch; 2 Federal Food Safety and Veterinary Office (FSVO), Risk Assessment Division, 3003 Bern, Switzerland; hans.reinhard@blv.admin.ch (H.R.); otmar.zoller@blv.admin.ch (O.Z.); 3 Biologisches Institut für Pollenanalyse K. Bieri GmbH, Talstrasse 23, 3122 Kehrsatz, Switzerland; katharina.bieri@pollenanalyse.ch

**Keywords:** pollen, bee bread, pollen analysis, *Echium vulgare*, pyrrolizidine alkaloids, echivulgarine

## Abstract

Previous studies have shown that pollen products sold as nutritional supplements and used in apitherapy may contain toxic pyrrolizidine alkaloids (PAs) if bees collect pollen from PA-containing plants, such as *Echium vulgare*. In this study, the botanical origin of pollen from two observation sites was studied. Despite a high PA content in pollen samples that bees collected during *E. vulgare*’s flowering period, bees were found to collect relatively few *Echium* pollen loads. Thus, the monitoring of pollen loads collected at the apiaries is unviable to estimate the risk of PA contamination in pollen or bee bread. In a second step, the stability of PAs in bee bread samples containing PAs at concentrations of 2538 ng/g and 98 ng/g was assessed over a period of five or six months, respectively. No significant PA reduction was observed in bee bread stored at 15 °C, but there were overall PA reductions of 39% and 33% in bee bread stored at 30 °C, reflecting hive conditions. While PA *N*-oxides decreased over time, other types of PAs remained relatively stable. Monitoring PAs in pollen products remains important to ensure consumer safety and should include echivulgarine (and its *N*-oxide), the major PA type found in pollen from *E. vulgare*.

## 1. Introduction

Honeybees collect pollen from the anthers of flowers, adding nectar or honeydew to form pollen loads. These loads are brought back to the hive, where they add salivary gland secretions. Loads are then stored in the hive’s combs, where lactobacillus bacteria ferment the pollen to form bee bread [1]. Beekeepers collect pollen loads with traps installed at the entrance of the beehives, and they collect bee bread directly from the combs. Although bee bread harvesting is more complex, bee bread is more digestible than pollen, and its nutrients and biologically active compounds are more easily absorbed [2].

Because both pollen and bee bread contain significant quantities of vitamins and minerals and are rich in biologically active compounds, such as polyphenols and flavonoids [2,3], they are used as dietary supplements or as antioxidants, antimicrobials, or anti-inflammatory agents [2,3,4,5,6]. The use of bee-collected pollen in nutritional supplements or for apitherapy has prompted a number of research studies, investigating its composition and therapeutic use as well as its quality and safety. The latter studies include residue analysis to detect contaminants from beekeeping or the environment [7] and analysis for phytotoxins related to the pollen’s botanical origin [3,5].

Previous studies have reported that the pollen that bees collect from pyrrolizidine alkaloids (PA)-producing plants can contain toxic PAs [8,9,10,11,12,13,14,15,16]. This is a critical concern for food safety and/or therapeutic applications, as PAs can cause liver and lung damage or cancer [17,18,19,20,21]. PA-producing plant species belong mainly to the families Asteraceae (tribes Senecioneae and Eupatorieae), Fabaceae (genus *Crotolaria*) and Boraginaceae (all genera) [22,23]. In European bee products, the most frequently detected PAs are those belonging to the genus *Echium* [12,13,15,24]. In Switzerland (as in most European countries), the main *Echium* species is *Echium vulgare*. Its pollen has a very high PA content, and echivulgarine *N*-oxide is the most prominent type [25].

The aim of this study was to expand existing knowledge of PAs in pollen or bee bread of European origin in order to provide advice to beekeepers in relation to required quality procedures. In many cases, echivulgarine (and its *N*-oxide) is not included in routine control analysis, and beekeepers who produce pollen and/or bee bread have to find a laboratory that offers analysis for a pollen specific PA spectrum, covering the major PAs found in pollen from *E. vulgare*.

For this reason, it seemed useful to investigate when bees collect PA-containing pollen loads that originate from *E. vulgare*. By monitoring the appearance of *Echium* pollen, beekeepers can determine when to discontinue pollen collection to avoid PA contamination or to assess the risk for bee bread production. The study also explored the stability of PAs in bee bread over several months, as the naturally occurring fermentation of bee bread with lactic acid bacteria may alter the PA content of bee bread. If the PAs in bee bread decrease very rapidly over time, it may not be necessary to determine PA content for quality control.

## 2. Results

### 2.1. Botanical Composition of Bee-Collected Pollen from Two Apiaries in the Southern Alps of Switzerland

The study’s principal aim was to examine the various pollen types that bees collect during the flowering period of *E. vulgare*. In previous work, we chose two sites in the Verzasca Valley, where *E. vulgare* is abundant and where beekeepers produce honey containing *Echium*-type PAs [24,26]. From the end of April until September (2012 to 2014), beekeepers collected pollen on one day per week, and pollen loads were subsequently sorted by color to determine pollen types.

**Figure 1:**
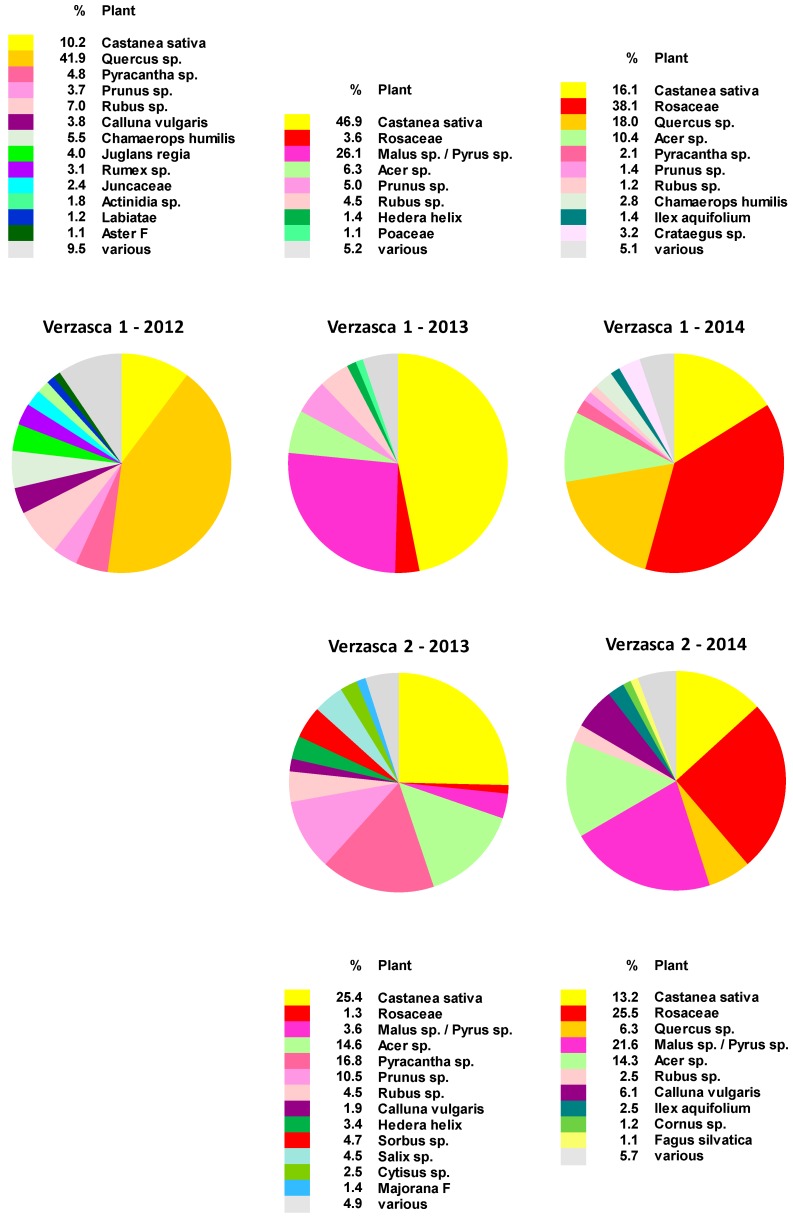
Main pollen types collected at two apiaries in the Verzasca valley during the flowering period of *Echium vulgare*. In Verzasca 1, 22 pollen samples were collected between 28 April and 22 August 2012, 75 samples were collected between 26 April and 21 September 2013, and 52 samples between 24 April and 15 August 2014. In Verzasca 2, 61 samples were collected between 23 April and 25 September 2013, and 52 samples between 22 April and 27 August 2014. The colors represent all pollen types exceeding 1% of the total amount of pollen collected per apiary per year. *Echium*-type pollen was not included in the main pollen types, as it accounted for less than 1%. All pollen types collected at the apiaries, as well as examples of pollen types in the group classifications Aster F, Majorana F, or in family classifications, are listed in Table S1 in Supplementary Materials.

Microscopic analysis revealed up to 74 different pollen types (Table S1 in Supplementary Materials), of which up to 13 types accounted for more than 1% each of the total pollen collected per year and location (Figure 1, Table S1). *Castanea sativa* pollen was consistently present and was among the most important pollen sources for bees during the observation period, accounting for between 10% and 47% of the total pollen collected each year. Other important pollen sources included tree species such as *Acer* sp., especially in 2013 and 2014, and *Quercus* sp., mainly in 2012 and 2014. Several Rosaceae pollen types were also regularly found, including *Rubus* sp., *Malus* sp., *Pyrus* sp. and *Pyracantha* sp., as well as pollen from other plants belonging to the Rosaceae family (e.g., *Amelanchier* sp. or *Cotoneaster* sp.), to which a genus level could not be assigned.

We previously analyzed some of these samples for PAs and reported *Echium*-type PAs up to 4584 ng/g (Table S2), mainly in samples collected during the month of July [15]. However, only a few *Echium*-pollen loads were present in samples collected in June and July, the main blooming season of *E. vulgare*, despite the high levels of *Echium*-type PAs. The all-years sum of *Echium*-type pollen was below 0.02% (Table 1 and Table S1).

### 2.2. Botanical Composition of Bee-Collected Pollen from an Apiary North of the Alps

In previous work, we also chose an additional site in Basel where *E. vulgare* was abundant and where a beekeeper produced commercial pollen containing *Echium*-type PAs [15,24]. The beekeeper collected pollen samples in similar fashion from April to September of 2012 and 2013.

**Figure 2:**
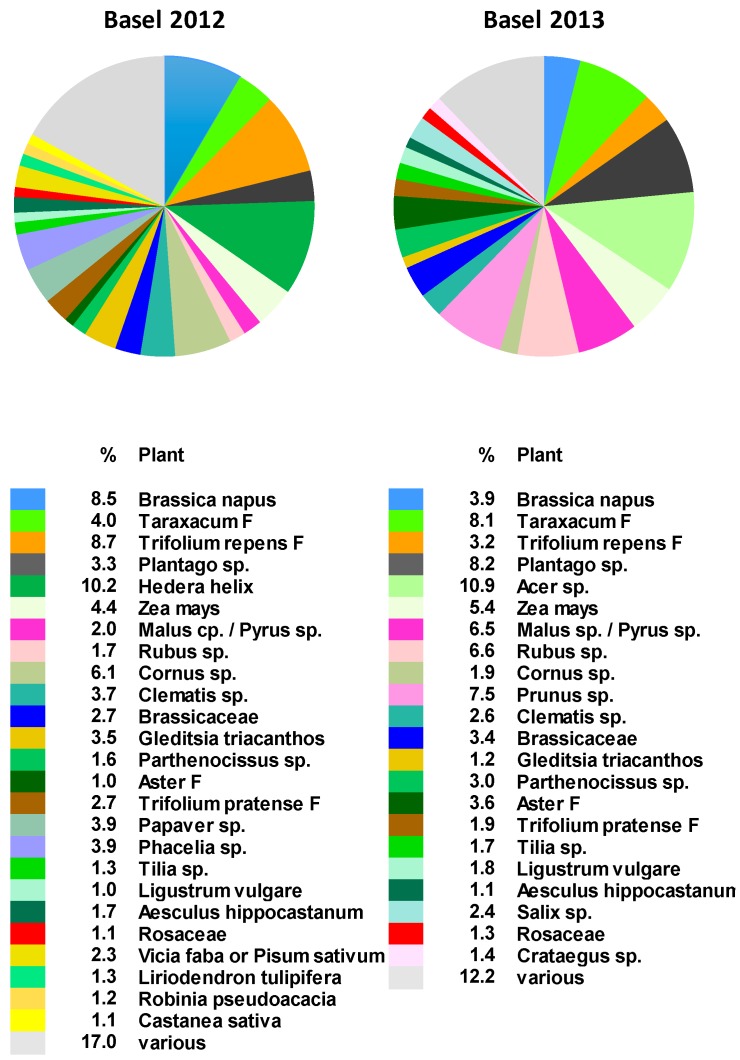
Main pollen types collected in Basel during the flowering period of *E. vulgare.* In total, 87 pollen samples were collected between 30 April and 30 September 2012, and 92 samples were collected between 22 April and 30 September 2013. The colors represent all pollen types exceeding 1% of the total pollen collected per apiary and year. *Echium*-type pollen was not included in the main pollen types, as it accounted for less than 1%. All pollen types collected at the apiaries, as well as examples of pollen types in the group classifications Aster F, Taraxacum F, Trifolium repens F, and Trifolium pratense F, are provided in Table S1 in Supplementary Materials.

The pollen spectrum from Basel was more diverse than that obtained from the Verzasca valley. The analysis revealed up to 134 different pollen types (Table S1 in Supplementary Materials), among which up to 25 pollen types accounted for more than 1% each of the total pollen collected per year (Figure 2, Table S1).

For the northern part of Switzerland, the main pollen types [27] were typically meadow plants, such as *Taraxacum* F (mainly *Taraxacum officinalis*), *Trifolium repens* F, and *Plantago* sp., as well as cultivated plants, such as *Brassica napus* and *Zea mays.* The other main pollen sources were *Acer* sp., *Malus* sp., *Pyrus* sp., *Rubus* sp., *Cornus* sp., and *Hedera helix*.

Total *Echium*-type pollen was 0.012% in 2012 and 0.001% in 2013 (Table 1 and Table S1). Although we had previously found *Echium*-type PAs, especially in samples collected in June and July 2012 [15] (Table S2), only a few *Echium* pollen loads were found in these samples.

### 2.3. Stability of PAs and PA N-oxides in Bee Bread

**Figure 3:**
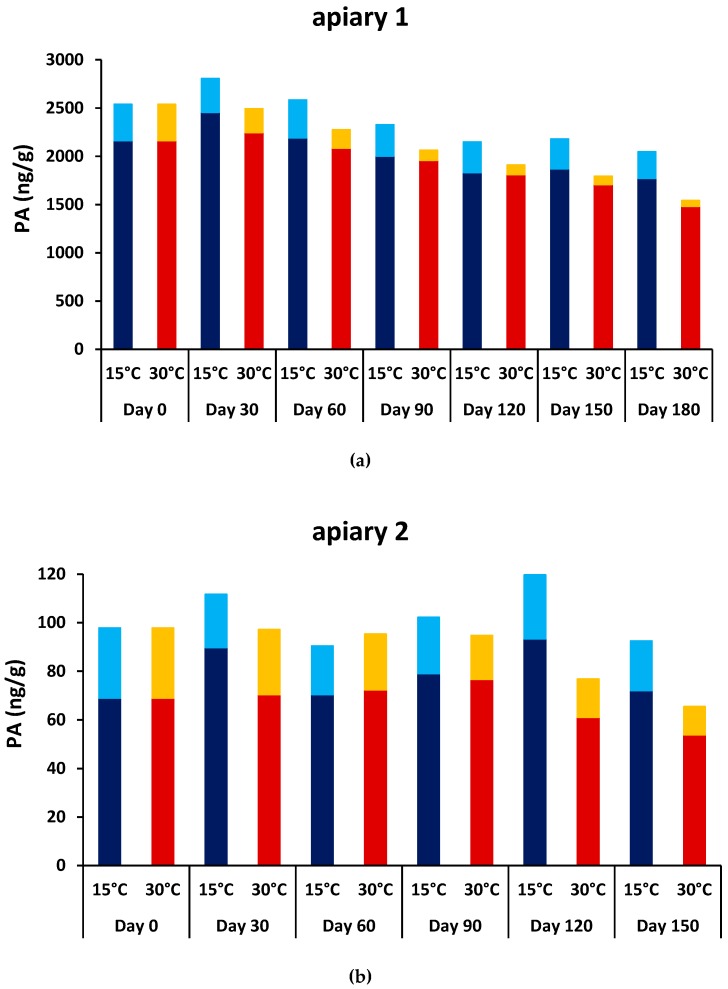
Concentrations of total pyrrolizidine alkaloids (PAs) in bee bread over storage periods of five and six months for (**a**) apiary 1 and (**b**) apiary 2, respectively. Tertiary PAs (sum of PAs and acetyl-PAs) in bee bread stored at 15 °C (dark blue) or at 30 °C (dark red) and sum of PA *N*-oxides (sum of PA *N*-oxides and acetyl-PA *N*-oxides) in bee bread stored at 15 °C (light blue) or at 30 °C (orange). Bee bread samples were determined in duplicates and are presented as averages. Since standards were not available for all PA-types, values are best-estimates.

In the second step, we investigated whether PAs were stable in bee bread over a period of half a year. To that end, we monitored PA content over time at two different temperatures: 30 °C (reflecting conditions inside a beehive where bee bread is stored) and 15 °C (reflecting storage conditions outside the hive). PA-containing bee bread was collected from two apiaries. At apiary 1, *E. vulgare* was flowering in large quantities on the roof of a large building close to the beehives; no *E. vulgare* plants were observed at apiary 2. At harvest (Day 0), the bee bread from apiary 1 contained PAs at substantially higher concentrations (total PA = 2538 ng/g) than the bee bread from apiary 2 (total PA 98 ng/g) (Figure 3). The bee bread from apiary 1 mainly contained PAs typically found in the pollen of *E. vulgare* [25,28], such as echivulgarine (*N*-oxide), vulgarine, and echimidine, as well as intermedine congeners, which are PAs that may originate from *Eupatorium cannabinum* (Table 2). In the bee bread from apiary 2, intermedine congeners were more prominent than echivulgarine (*N*-oxide) (Table 2). In total, we detected 17 PAs and PA *N*-oxides (Table 2). Interestingly, nearly all detected PA/PA *N*-oxides belonged to the mono- or diester-type, with the exception of very low amounts of macrocyclic diester PA/PA *N*-oxides (i.e., retrorsine, retrorsine *N*-oxide, and senecionine) in the bee bread from apiary 2. Along with the identification criteria listed in Table 3, and as expected for PA *N*-oxides, weak to moderate dimer ions (2MH+) were observed. Acetyl-PA-types were relatively rare (2–3%) in the sample from apiary 1 but accounted for 35–71% of the sample from apiary 2.

As shown in Figure 3, overall PA content remained relatively stable over a period of six months, especially for bee bread samples stored at 15 °C. Upon storage at 30 °C, the observed overall PA fell by 39% and 33% in the bee bread from apiary 1 and apiary 2, respectively, due mainly to a decline in PA *N*-oxides (Figure 3). However, PA *N*-oxides did not disappear completely during the test period. Other PA-types remained relatively stable, other than a small intermediate rise in acetyl-PA in bee bread from apiary 2. Taken together, no significant PA reduction was observed in the bee bread stored at 15 °C, while there was some reduction in the bee bread stored at 30 °C.

## 3. Discussion

As pollen loads collected during *E. vulgare*’s flowering period that originated from *E. vulgare* were very sparse, monitoring pollen types proved unsuitable as a means of assessing the risk of PA contamination of pollen products. Furthermore, the majority of PAs remained stable in the bee bread samples during storage over several months. Hence, monitoring PAs remains an important element of ensuring good quality of pollen products. Chemical analysis should include the essential PA spectrum, covering the major PAs found in pollen from *E. vulgare*.

### 3.1. Pollen Spectrum in the Northern and Southern Parts of Switzerland

In Basel, the main pollen types collected from April to September included agricultural crops, such as corn (*Zea mays*), rape (*Brassica napus*), and white clover (*Trifolium repens*), as well as pasture plants, such as dandelion (*Taraxacum officinalis*) and plantain (*Plantago* sp.), and various tree species, such as maple (*Acer* sp.), pome, and stone fruits (*Malus* sp., *Pyrus* sp., *Prunus* sp.). These plant taxa were already among the main pollen sources for bees 40 years ago and are commonly found in the northern part of Switzerland [27] and in other European countries [29].

Weather conditions at a given location can differ considerably in consecutive years, influencing the availability of various pollen sources for bees at a given time, and the amount of a particular pollen type collected may therefore differ substantially from year to year. This can be seen in the varying amounts of a typical pollen spectrum for southern Switzerland [27,29], consisting of oak (*Quercus* sp.), maple (*Acer* sp.), and various *Rosaceae*, including berries (*Rubus* sp.) and pome or stone fruits (*Malus* sp., *Pyrus* sp., *Prunus* sp.). On the other hand, *Castanea sativa*, the European sweet chestnut, was consistently among the most important pollen sources for bees, as already documented in earlier studies for southern Switzerland [29]. During the years 2012 to 2014, chestnut trees across the entire region were severely affected by the Asian chestnut gall wasp, resulting in greatly reduced flower production [30]. Despite the damage caused by that attack, the pollen of *Castanea sativa* remained a very important pollen source for bees during our observations in the Verzasca valley. We noted that bees collected large quantities of *Castanea sativa* pollen mainly during the month of June, when *E. vulgare* and the chestnut trees were blooming simultaneously. The total quantities of bee-collected pollen fell drastically when chestnut pollen was no longer available. Interestingly, *Echium*-type PAs appeared mainly in pollen collected in July [15], suggesting that bees collected *Echium* pollen in the absence of any more abundant or attractive pollen sources, such as *Castanea sativa*.

Despite their high PA content, the July bee-collected pollen samples contained surprisingly few *Echium* pollen loads. One possible explanation is that *E. vulgare* pollen contains very high concentrations of PA, ranging from 5.4 mg/g (Verzasca 2013) to 24.5 mg/g (Basel 2014) at our observation sites [25]. This means that just a few pollen loads suffice to contaminate overall pollen production. When calculating with 20 mg/g, even as low as 0.005% of *E. vulgare* pollen leads to 1000 ng/g PA in the pollen product. It follows that beekeepers who produce pollen products should choose their apiary locations carefully, avoiding large fields of PA-containing plants in the immediate vicinity.

### 3.2. Tertiary PAs and Corresponding PA N-oxides in Plants, Pollen, and Bee Bread

In plants, PAs usually occur as PA *N*-oxides rather than the corresponding free bases (tertiary PAs) [22,31]. For example, *E. vulgare* flower heads mainly contain *Echium*-type PA *N*-oxides, while the corresponding tertiary PAs are almost negligible [15]. Similarly, *Senecio* sp. or *Eupatorium cannabinum* flower heads mainly contain *Senecio*- or *Eupatorium*-type PA *N*-oxides [15]. The same is true for plant pollen [8,25,28]. Although less prominent, PA *N*-oxides are usually more prevalent than the corresponding tertiary PAs in bee-collected pollen [9,13,15]. However, storage conditions may affect the ratio of PA *N*-oxides to tertiary PAs in bee-collected pollen, as heating or prolonged storage is likely to reduce PA *N*-oxides [12].

In contrast to plants or bee-collected pollen, tertiary PAs were dominant in freshly harvested bee bread, accounting for 85% (apiary 1) and 71% (apiary 2) of the total PA content. Additionally, PA *N*-oxides diminished with prolonged storage time, but tertiary PAs did not. The fall in PA *N*-oxides was faster at an elevated temperature of 30 °C and very modest at 15 °C. Figure 3 shows this effect for both analyzed samples. While time-dependent reduction of PA *N*-oxides is clearly visible for bee bread from apiary 1, with a high initial total PA content, the reduction is less obvious for bee bread from apiary 2, which had a lower initial total PA content. In this case, PA and PA *N*-oxide detection was hindered by few signal intensities around the detection limit, leading to elevated imprecision and scatter.

This reduced stability of PA *N*-oxides during storage has previously been reported for honey [32,33]. While tertiary PAs remained stable during six months of storage, PA *N*-oxides in honey decreased within a few days. As the content of tertiary PAs did not increase accordingly, a simple reduction of PA *N*-oxides to corresponding tertiary PAs can probably be excluded. Interestingly, and contrary to the honey study, PA *N*-oxide reduction was slower in bee bread, and the PA *N*-oxides in our bee bread samples did not vanish completely over several months of storage. PA *N*-oxide reduction might be caused by digestive enzymes that the bees added to the honey or bee bread [32,33]. However, the mechanism of PA *N*-oxide -breakdown, which is not yet completely understood in honey, may not be the same for bee bread. In summary, more than half of the initial bee bread PAs were retained at storage temperatures reflecting hive conditions (30 °C). It follows that monitoring of PAs remains important for bee bread quality control.

### 3.3. Recommendations for Maximum PA Content in Pollen Products

Performing a reliable risk assessment for PAs remains difficult, and this situation is unlikely to improve substantially in the near future. Non-carcinogenic effects are not discussed here because they are only of concern at higher intakes. According to the opinion of the European Food Safety Authority (EFSA) of 2011 [23] the intake of 7 ng/kg body weight (bw) per day is of low concern for an excess lifetime cancer risk (margin of exposure (MOE) of 10,000 and using a benchmark dose lower confidence limit for a 10 % excess cancer risk (BMDL_10_) of 70 µg/kg bw per day for the induction of liver haemangiosarcoma by lasiocarpine in male rats as a reference point). There are substantial uncertainties in this assessment. EFSA adapted its reference value with a statement in 2017 [34] to 24 ng/kg bw per day, using a different reference point and model averaging (MOE of 10,000 and using a BMDL_10_ of 237 µg/kg bw per day, derived for the incidence of liver haemangiosarcoma in female rats exposed to riddelliine as a reference point). If one tries to calculate thereof an acceptable concentration for pollen or bee bread, one should consider that there are other sources of PAs, like honey, tea, herbal tea, and all products containing herbs. On that basis, the allocation to pollen products should be 50% at maximum, perhaps more ideally 20%. Assuming an intake of 5 g of pollen or bee bread per day, a bw of 60 kg and an allocation of 20%, a concentration of total PA/PA *N*-oxides of 60 ng/g might be acceptable. A 50% allocation would lead to a concentration of 140 ng/g. Infants and toddlers should not consume pollen products; if children consume pollen or bee bread, the respective concentrations should be correspondingly lower. We would also like to point out that the ALARA (as low as reasonably achievable) principle should always be applied for genotoxic and carcinogenic substances.

### 3.4. PA Content in Pollen and Bee Bread

Pollen products contain PAs more frequently and often at higher concentrations than honey [10,12]. In PA-positive pollen samples (31% respectively 60%), average PA concentrations were 5170 ng/g and 1846 ng/g [10,12], and a few samples exhibited PA levels several hundred times higher than recommended. Such products are not suitable for food or therapeutic applications. Other studies have reported lower PA levels. In 11 of 12 tested pollen products of European origin, PAs were detected, with a mean concentration of 576 ng/g [13]. We previously reported that 31% of tested commercial pollen samples (*n* = 32) produced in Switzerland were PA positive, with overall average PA concentrations of 100 ng/g and 319 ng/g for positive samples [15]. It seems likely, then, that PA frequencies and levels vary substantially according to the pollen’s geographical origin. However, variations are also likely to depend on the chosen analytical method; a sample’s PA content may be underestimated if the analysis does not take account of the entire PA spectrum for pollen (e.g., if echivulgarine (*N*-oxide) is not included for pollen originating from *E. vulgare*).

To date, information about PAs in bee bread is sparse. We studied eight bee bread samples collected from five different apiaries during 2017 and 2018. Among these were three samples (produced in 2017) obtained after one year of storage, three samples produced in 2018 and two freshly harvested samples (2018). The long-term studies described here involved only freshly harvested samples. Bee bread collected from apiary 1 contained PAs at levels (2538 ng/g) about 20 to 40 times the recommended maximum concentrations of 60 or 140 ng/g, respectively, while the PA content of the bee bread from apiary 2 (98 ng/g) was around the maximal acceptable PA level. One further sample had a PA-content of 436 ng/g, which is also above the recommended level; the other five samples exhibited low PA levels, ranging from 9 to 63 ng/g (data not shown). The most prominent analytes in these additional samples were intermedine (*N*-oxide)-type and acetyl-intermedine-type PAs and echivulgarine. Two samples also contained seneciphylline (*N*-oxide) (data not shown). It is therefore important to monitor a wide range of PAs for quality surveillance of both pollen and bee bread.

### 3.5. Untargeted Analysis to Determine PAs in Bee Bread

As a further aim of this research was to identify not only known but also unknown PA/PA *N*-oxides in bee bread, it was necessary to combine full-scan mass spectrometric detection with mass fragmentation information. Additionally, the acquisition of full-scan spectra allowed for post-analysis data mining. The chosen instrument fulfilled these requirements, but one drawback was its detection sensitivity for PAs of 1 ng/g—a factor of 10 lower than for a state of the art triple-quadrupole instrument operated in selected reaction monitoring-mode.

We are conscious that the values derived from our quantitation method, which compared the analyte’s content to an internal standard, must be considered an estimate. Based on our experience of other PA/PA *N*-oxide -containing matrices, we are aware that PA/PA *N*-oxides would ideally be quantified by means of a standard addition procedure, but this is feasible only for quantification of PAs for which the reference substances are available. Here, we relate all PAs to D3-atropine. For most available PA references, the detector response factors were similar to the response of D3-atropine. Since no references were available for echivulgarine *N*-oxide or vulgarine, their response factors could not be determined. Detector response correction was applied where possible, accounting for 83% and 65% of the total PA-content in bee bread of apiary 1 and 2, respectively. In our opinion, such an estimation is adequate for studying the course of PA reduction over time, especially since a precise quantification is not possible as long as some of the references are not available.

Here, as in other PA/PA *N*-oxide-containing matrices, it is important to include the contribution of the major PA/PA *N*-oxides, independent of the availability of the corresponding reference substances. Most importantly, our method included echivulgarine (*N*-oxide), which is the most abundant PA type in *E. vulgare* pollen [15,25,35]. In contrary, targeted methods often do not account for echivulgarine and echivulgarine *N*-oxide, since references are not commercially available.

## 4. Materials and Methods

### 4.1. Collection of Pollen Samples from Two Observation Sites

The first site was in the Verzasca valley, south of the Alps in the southern part of Switzerland. Two beekeepers participated in the project [15], with apiaries approximately 400 m apart (beeline distance). The second site was north of the Alps, near Basel in northern Switzerland. At the Verzasca observation sites, traps were attached in front of the beehive entrances; in Basel, the pollen traps were integrated in the beehive systems. When weather conditions permitted, the beekeepers collected pollen one day (24 h) per week from two to four colonies during the months of April to September, as previously described [15]. The traps were emptied at the end of the day, and samples were immediately frozen at -20 °C. Samples were dried at 30 °C for 48 h prior to the analysis of pollen types present in each sample. In total, 262 pollen samples were taken from the Verzasca valley (Verzasca 1: *n* = 22 (2012); *n* = 75 (2013); *n* = 52 (2014). Verzasca 2: *n* = 61 (2013); *n* = 52 (2014)), and 179 were taken from Basel (*n* = 87 (2012); *n* = 92 (2013)).

### 4.2. Determination of Botanical Origin

For each sample, 3 g of pollen was taken for analysis; for small sample sizes below 3 g, the entire sample was taken. Pollen pellets were divided into fractions according to color and shape [36]. For the analysis, small parts were removed from pollen loads, and the pollen was mixed in a drop of preheated mounting medium [37] on a microscope slide and secured with a cover slide prior to microscopy. Fractions were combined if they contained the same pollen types. For quantification, the weight of each fraction was determined. This way, the botanical composition of each sample was obtained. To obtain an average pollen composition per day, data from all samples collected on the same day at the same observation site were then combined and divided by the number of samples (from different colonies).

The data were summed for each location and collection period (April to September) and expressed as the amount in grams of a specific pollen type collected during the observation period (averaged per colony). The occurrence of each pollen type was also expressed as a percentage of the total amount of pollen collected during the observation period (averaged per colony).

A few pollen types had distinctive morphologies that enabled identification at species level (e.g., *Castanea sativa*). However, pollen types were usually determined at genus level (e.g., *Prunus* sp. or *Rubus* sp). If this was not possible, pollen types were determined at family level (e.g., Rosaceae, which included *Amelanchier* sp. and *Cotoneaster* sp.) (Table S1, Supplementary Materials). Some pollen types were determined at the family group level (e.g., Aster F) (Table S1, Supplementary Materials).

### 4.3. Collection of Bee Bread

Bee colonies (*Apis mellifera*) were located in Bern, Switzerland (apiary 1, 10 colonies) and in Witzwil, Switzerland (apiary 2, 12 colonies). Colonies were on ten frames in 12-frame Dadant Blatt hives. Bee bread was collected on 23 August 2018 (apiary 1) and on 25 September 2018 (apiary 2) from core frames of three colonies in each apiary. Bee bread pellets were removed from single cells using a spatula, avoiding as far as possible the collection of beeswax. Finally, 100 g of bee bread from each apiary was homogenized manually in a mortar to minimize mechanical destruction of the matrix and to maintain the utmost biochemical activity of the matrix. However, this manual homogenization was achieved at the expense of increased data scatter, especially for the sample from apiary 2, where the PA/PA *N*-oxide -content was low and close to the detection limit. The homogenized samples were subsequently divided into two batches, one stored at 15 °C and the other stored 30 °C, for five and six months, respectively.

### 4.4. Reagents and Materials for liquid chromatography mass spectrometry (LC-MS)

A mixture of 7-acetyl-intermedine/lycopsamine was from PlantaAnalytica (Danbury, US), retrorsine from Cfm O.Tropitzsch (Marktredwitz, Germany), and echimidine, intermedine, intermedine *N*-oxide, and senecionine were from PhytoLab (Vestenbergsgreuth, Germany). Echivulgarine was isolated in-house [38] and echimidine *N*-oxide and retrorsine *N*-oxide were *N*-oxidized in-house, starting from the free bases according to [39], except that dichloromethane was used instead of trichloromethane.

D3-atropine was from CDN isotopes (Quebec, Canada), methanol was from Honeywell (LC-MS grade, Grogg AG, Stettlen, Switzerland). Sulfuric acid, formic acid, and aqueous ammonia solution 25%, all of analytical grade, were from Merck (Grogg AG, Stettlen, Switzerland). Ultrapure water was used (Elga purelab ultra, Labtec, Villmergen, Switzerland).

### 4.5. Sample Preparation for LC-MS

For the analysis, aliquot samples were taken either from the freshly homogenized material or from samples stored at 15 °C and 30 °C for 30, 60, 90, 120, 150, and 180 days. A total of 1–2 g of bee bread was transferred to a centrifuge tube, 20 µL of internal standard (D3-atropine in methanol, c = 10ng/mL), and 20 mL 0.05 M aqueous sulfuric acid solution were added, and the mixture was sonicated for 15 min. The suspension was passed to a 25 mL-spin filter tube (0.45 µm, Nylon, FisherScientific, Reinach, Switzerland) and centrifuged at 2200 *g* for 15 min. The filtrate was passed to a solid-phase extraction SCX cartridge (SampliQ, Agilent, MSP Kofel, Zollikofen, Switzerland), previously conditioned with 3 mL methanol and 3 mL 0.05 M aqueous sulfuric acid solution by an Aspec GX-274 robot (Gilson, Mettmenstetten, Switzerland). After washing the loaded column with 3 mL 0.05 M aqueous sulfuric acid solution, followed by 3 mL water and 5 mL methanol, the analytes were eluted with 8.5 mL ammonia in methanol (1.5% *v*/*v*), and the solvent evaporated to near dryness under nitrogen at 40 °C. Samples were dissolved to an end volume of 0.5 mL in methanol/water/formic acid (50/50/0.1%, *v*/*v*/*v*).

### 4.6. Identification and Quantification of PA/PA N-oxides in Bee Bread by LC-MS

A liquid chromatography (LC) system equipped with a degasser, autosampler, controller, two high-pressure gradient pumps, static mixer, and a column oven (LC20, Shimadzu, Reinach, Switzerland) was linked to a time-of-flight (TOF) mass spectrometer (Maxis 4G+, Bruker, Fällanden, Switzerland). This instrument is of quadrupole-collision cell-reflectron-TOF-geometry and supports high-resolution mass detection, selected and unselected mass fragmentation, and software-aided targeted and non-targeted compound screening.

Analyte separation was accomplished on a Kromasil C18 analytical column (125 × 2 mm, 3.5 µm particle size, Macherey-Nagel, Buchs, Switzerland). A solvent system methanol/0.1% formic acid (*v*/*v*, solvent B)—water/0.1% formic acid (*v*/*v*) was used. A total of 5 µL of sample were injected at 10%B, a flow rate of 0.2 mL/min and 30 °C column temperature. After 1 min, a linear gradient to 40%B was applied, within 6 min, followed by a linear gradient to 90%B, within 6 min. Elution then proceeded isocratically for 5 min, followed by a linear gradient to 10%B over 2 min and equilibration at 10%B for 2 min.

The mass spectrometer (MS) was operated in positive electrospray ionization-mode (ESI). The scanned mass range was 50–1000 amu, resolution 35,000 at 400 amu, spectra rate 1 Hz, unselected precursor fragmentation MS/MS-rate 3 Hz, with 35 eV collision energy and nitrogen as the collision gas. Source parameters were plate offset 500 V, capillary voltage 4.5 kV, dry temperature 200 °C, nebulizer gas pressure 150 kPa and nitrogen dry gas flow rate 8 L/min. Sodium formate was used as the calibrant. Compass 1.7 and TargetAnalysis 1.3 were used for instrument control and data analysis, respectively.

Identification of PA/PA *N*-oxides was based on comparing retention times, high-resolved and fragment masses from MS/MS experiments, either to standard substances or the literature [28,40].

Quantification was carried out by using extracted ion chromatograms at accurate masses. The peak resolution was constant for all analytes (35,000) in the chosen mass range. The applied internal standard calibration ensured adequate recovery-correction with respect to the extraction procedure. We used D3-atropine as the internal standard (IS), since it has a similar mass (MH+, *m*/*z* = 291), retention time (Rt = 10.0), detection response, and belongs to the same chemical class as the PAs under investigation. Detector responses were similar to the response of D3-atropine for 7 out of 11 available PA references. For details see Figure S1 in Supplementary Materials. The data for available PAs were corrected for detector response. The concentration of unknowns was calculated as c(unknown) = peak_area(unknown)/peak_area(IS) x c(IS). The limit of detection (LOD) was 1 ng/g.

## 5. Conclusions

Our study showed that monitoring *Echium* pollen loads by pollen analysis is not suitable to determine a harvest time-window for production of PA-free pollen products. Furthermore, most PA types remained stable over several months in bee bread. Therefore, we conclude that the PA content of pollen products should be monitored by chemical analysis to ensure good quality. The chosen analytical method should include echivulgarine (*N*-oxide), which is the most abundant PA type in *E. vulgare* pollen. As targeted methods often do not include echivulgarine (*N*-oxide), it seems likely that PA levels in pollen of European origin are considerably underestimated.

## Figures and Tables

**Table 1:** *Echium*-type pollen content at two apiaries in the Verzasca valley and in Basel.

Location	Year	*Echium*-Type Pollen	
		% of Total Pollen	(g)
Verzasca 1	2012	0.003	0.01
Verzasca 1	2013	0.013	0.04
Verzasca 1	2014	0.004	0.01
Verzasca 2	2013	0.004	0.01
Verzasca 2	2014	0.005	0.02
Basel	2012	0.012	0.10
Basel	2013	0.001	0.01

**Table 2:** PA-types and minimum and maximum concentrations of PAs in bee bread during a storage period of six (apiary 1) or five months (apiary 2) at 15 °C or 30 °C.

PA-Types	PA Conc. Range in Bee Bread from Apiary 1 (ng/g)	PA Conc. Range in Bee Bread from Apiary 2 (ng/g)
Intermedine-type	116–221	28–56
Intermedine *N*-oxide -type	nd–62	nd–3
Retrorsine	nd	nd–2
Retrorsine *N*-oxide	nd	nd–2
7-Acetyl-intermedine-type	nd–10	nd–11
Senecionine	nd	nd–2
Echimidine *N*-oxide	2–26	nd–1
Di-acetyl-intermedine-type *	1–13	nd–14
Di-acetyl-intermedine *N*-oxide -type *	7–18	12–26
Echimidine	98–167	2–5
Vulgarine *	65–118	nd
Vulgarine *N*-oxide *	7–36	nd
Acetyl-echimidine/vulgarine *	8–19	nd–1
Acetyl-echimidine *N*-oxide *	3–6	nd–1
Acetyl-vulgarine *N*-oxide *	3–13	nd
Echivulgarine	1176–1950	8–36
Echivulgarine *N*-oxide *	32–248	nd–2

* Tentative identification according to retention time and specific fragment masses; values are related to the internal standard (IS) and are therefore best-estimates; nd: not detected (<LOD). The term *type* comprises all isotopic isomers, which could not be fully separated by this chromatographic method. Bee bread samples were analyzed in duplicates and are presented as averages.

**Table 3:** Identification of pyrrolizidine alkaloids found in bee bread by high resolution mass spectrometry.

Pyrrolizindine Alkaloid	Sum Formula	Rt (min.)	Quantifier MH+	Qualifiers
Intermedine-type	C15H25NO5	7.2–7.5	300.1805	156.1020, 138.0913, 120.0808
Intermedine *N*-oxide-type	C15H25NO6	7.6–8.2	316.1755	172.0968, 155.0941, 136.0757
Retrorsine	C18H25NO6	8.8	352.1755	324.1806, 220.1331, 138.0913
Retrorsine *N*-oxide	C18H25NO7	8.9	368.1704	340.1755, 220.1331, 136.0757
7-Acetyl-intermedine-type	C17H27NO6	9.0	342.1911	198.1125, 180.1019, 120.0808
Senecionine	C18H25NO5	10.5	336.1805	308.1880, 220.1331, 138.0913
Echimidine *N*-oxide	C20H31NO8	11.2	414.2122	396.2017, 352.1755, 254.1387
Di-acetyl-intermedine-type *	C19H29NO7	11.3	384.2017	324.1806, 240.1230, 180.1019
Di-acetyl-intermedine *N*-oxide-type *	C19H29NO8	11.3	400.1966	340.1755, 180.1019, 136.0757
Echimidine	C20H31NO7	11.4	398.2173	238.1457, 220.1331, 138.0913
Vulgarine *	C20H31NO7	11.5	398.2173	380.2068, 254.1387, 240.1230
Vulgarine *N*-oxide *	C20H31NO8	11.8	414.2122	314.1598, 256.1179, 172.0968
Acetyl-echimidine/vulgarine *	C22H33NO8	12.3	440.2279	422.2173, 380.2068, 138.0913
Acetyl-echimidine *N*-oxide *	C22H33NO9	12.4	456.2228	438.2122, 338.1598, 220.1331
Acetyl-vulgarine *N*-oxide *	C22H33NO9	12.8	456.2228	438.2122, 298.1285, 180.1019
Echivulgarine	C25H37NO8	13.8	480.2592	380.2068, 322.1649, 220.1331
Echivulgarine *N*-oxide *	C25H37NO9	13.8	496.2541	396.2017, 338.1598, 220.1331

* Tentative identification according to retention time (Rt) and specific fragment masses. The term *type* comprises all isotopic isomers at indicated Rt.

## Supplementary Materials

The following data are available online at https://www.mdpi.com/1420-3049/24/12/2214/s1, Table S1: Microscopic analysis of pollen from Southern and Northern areas of Switzerland, Table S2: maximal total *Echium*-type PA concentrations in pollen samples, Figure S1: detector responses of PA references in comparison to D3-atropine as IS.
