# Supplementary material for: Pyrrolizidine Alkaloids: The Botanical Origin of Pollen Collected during the Flowering Period of Echium vulgare and the Stability of Pyrrolizidine Alkaloids in Bee Bread
Source: Molecules. 2019 Jun 13;24(12):2214. doi: 10.3390/molecules24122214 (PMC6631664; doi:10.3390/molecules24122214)
Supplement: Supplementary file 1 [file molecules-24-02214-s001.zip › Kast_Figure S1.pdf]

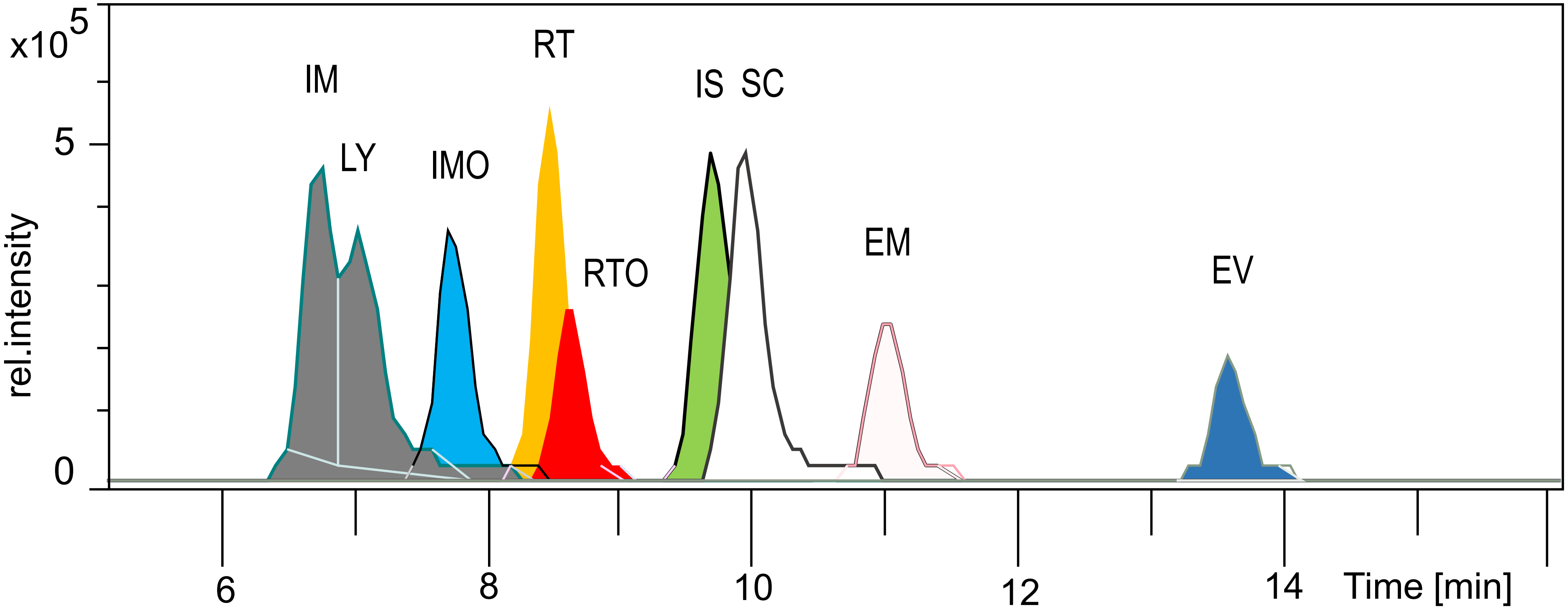

Figure S1: detector responses of PA references in comparison to D3-atropine as IS.
Response of standard-PAs: intermedine (IM), lycopsamine (LY), intermedine N-oxide (IMO), retrorsine (RT), retrorsine N-oxide (RTO), D3-atropine (IS), senecionine (SC), echimidine (EM) and echivulgarine (EV), 500pg on-column, each, solved in methanol (c=0.1µg/mL).
